# Magnetic resonance imaging for suspected perianal Crohn's disease in children: a multi-reader agreement study

**DOI:** 10.1007/s00330-025-11469-5

**Published:** 2025-03-23

**Authors:** Pradipta Debnath, Michael R. Acord, Christopher G. Anton, Jesse Courtier, Alexander M. El-Ali, Monica M. Forbes-Amrhein, Michael S. Gee, Mary-Louise C. Greer, R. Paul Guillerman, Murat Kocaoglu, Shailee V. Lala, Mitchell A. Rees, Gary R. Schooler, Alexander J. Towbin, Bin Zhang, Jason S. Frischer, Phillip Minar, Jonathan R. Dillman

**Affiliations:** 1 Department of Radiology, Cincinnati Children’s Hospital Medical Center, Cincinnati, OH USA; 2 Department of Radiology, Children’s Hospital of Philadelphia, Philadelphia, PA USA; 3 Department of Radiology, University of Cincinnati College of Medicine, Cincinnati, OH USA; 4 Department of Radiology and Biomedical Imaging, Benioff Children’s Hospital, San Francisco, CA USA; 5 Department of Radiology, NYU Langone Health, New York, NY USA; 6 Department of Radiology and Imaging Sciences, Riley Hospital for Children, Indianapolis, IN USA; 7 Division of Pediatric Imaging, Department of Radiology, Massachusetts General Hospital, Boston, MA USA; 8 Department of Diagnostic and Interventional Radiology, The Hospital for Sick Children, Department of Medical Imaging, University of Toronto, Toronto, Ontario Canada; 9 Department of Radiology, Nationwide Children’s Hospital, Columbus, OH USA; 10 Division of Biostatistics and Epidemiology, Cincinnati Children’s Hospital Medical Center, Cincinnati, OH USA; 11 Department of Pediatrics, University of Cincinnati College of Medicine, Cincinnati, OH USA; 12 Colorectal Center, Division of Pediatric General and Thoracic Surgery, Cincinnati Children’s Hospital Medical Center, Cincinnati, OH USA; 13 Division of Gastroenterology, Hepatology and Nutrition, Cincinnati Children’s Hospital Medical Center, Cincinnati, OH USA

**Keywords:** Abscess, Crohn's disease, Fistula, Magnetic resonance imaging, Pediatrics

## Abstract

**Objectives:**

We aimed to assess inter-radiologist agreement when interpreting pelvic MRI in children with newly diagnosed perianal Crohn’s disease (CD).

**Materials and methods:**

In this retrospective multi-reader study, we identified pediatric patients (< 18 years of age) who underwent a pelvic MRI examination for newly diagnosed perianal CD. Images were de-identified and uploaded to a cloud-based image platform for review by 13 fellowship-trained pediatric radiologists The reviewers assessed for the presence of a fistula and abscess, categorization of different imaging findings, and classification using the Parks and St James’ University Hospital systems. Fleiss’ kappa (κ) statistics and intra-class correlation coefficients (ICC) were used to measure inter-reader agreement, along with 95% confidence intervals (CI).

**Results:**

Forty-six patients were included in our study (median age = 13.0 years [IQR: 10.5 to 16.0 years]); thirty-five (76.1%) were boys. Most imaging features showed fair agreement (κ = 0.21 to 0.35). There was moderate agreement for categorical fistula length (κ = 0.42 [95% CI: 0.32 to 0.53]), involvement of the genitalia (κ = 0.45 [95% CI: 0.26 to 0.63]), and presence of an abscess/collection (κ = 0.52 [95% CI: 0.31 to 0.73]). Maximum abscess/collection length had good agreement (ICC = 0.81 [95% CI: 0.41, 1.00]). There was an almost equal split (yes vs. no: 50.7% vs. 49.3%) regarding whether postcontrast T1-weighted images added value compared to T2-weighted images alone across all radiologists and examinations.

**Conclusion:**

Inter-radiologist agreement when interpreting pelvic MRI for perianal CD in children is fair for most imaging features, with fewer features demonstrating moderate or good agreement.

**Key Points:**

***Question*** *Pelvic magnetic resonance imaging (MRI) is used for diagnosing and monitoring children with perianal Crohn's disease (CD). Limited information is known about inter-radiologist agreement.*

***Findings*** *Agreement between pediatric radiologists when interpreting MRI for perianal CD in children is only fair for most imaging features (κ = 0.21 to 0.35).*

***Clinical relevance*** *Understanding MRI inter-radiologist agreement is crucial to improve the reliability of pelvic MRI in children with perianal Crohn’s disease since it may affect patient management (e.g., surgery); further radiologist education and improved imaging feature definitions may help improve inter-radiologist agreement.*

## Introduction

Children with Crohn’s disease (CD) may develop inflammation of the perianal and/or perineal regions in addition to intestinal involvement [1]. Manifestations of perianal CD include fistulas, abscesses, inflammatory masses, fissures, and skin tags [2]. A recent study in adults has shown that 32% of patients with CD experience perianal disease within 10 years of diagnosis [3]. Another study by Adler et al reported that 30.1% of children with CD developed perianal involvement at 6 years after diagnosis [2]. Around 10% of newly diagnosed pediatric patients with CD have been shown to have perianal fistulas and/or abscesses at the time of diagnosis [4].

Magnetic resonance imaging (MRI) is able to produce high-quality images of the perianal region without ionizing radiation [5]. In part due to its excellent soft tissue image contrast resolution and field of view, pelvic MRI has been shown to have a higher accuracy when assessing complex fistulas compared to other modalities, such as endorectal ultrasound [6, 7]. The European Society of Gastrointestinal and Abdominal Radiology (ESGAR) consensus statement and American College of Radiology (ACR) appropriateness criteria indicate that pelvic MRI should be a first-line imaging test for patients with known or suspected perianal CD [8, 9].

Multiple prior studies have evaluated inter-radiologist agreement for the assessment of perianal CD in adults using MRI. For example, one study demonstrated substantial to almost-perfect inter-reader agreement for the presence of perianal fistula (κ = 0.79) and abscess (κ = 0.93), respectively [10]. Unfortunately, there is limited information with regard to inter-reader agreement for pelvic MRI in children with CD and known or suspected perianal disease. Identifying areas of agreement and disagreement is critical to understanding whether MRI is a reliable test in children, and if this test can be confidently used to diagnose, monitor disease progression, and act as a treatment endpoint in the clinic and in clinical trials. MRI features with relatively low agreement may be targets of radiologist education or improved radiologic definitions, or alternatively, such features may need to be removed from routine clinical use.

The primary objective of our study was to assess inter-radiologist agreement when interpreting pelvic MRI in children with newly diagnosed perianal CD. The agreement was evaluated for various imaging features as well as common classification systems.

## Methods

This retrospective study was approved by the Ethics Review Board at Cincinnati Children’s Hospital Medical Center with a waiver of informed consent granted. All study activities were conducted in a HIPAA-compliant manner.

### Study sample

Department of Radiology imaging reports were searched (Illuminate InSight v4.3, Softek Illuminate) to identify pediatric patients (< 18 years old) who had undergone a clinically indicated pelvic MRI examination for known or suspected perianal CD between January 2018 and December 2023. Our search identified studies with reports describing fistula, abscess, inflammatory mass, or complex perianal disease. In patients with multiple studies, we only included the initial study. Patients were excluded if they had a previous diagnosis of perianal CD or if their pelvic MRI examination did not include all four of the following pulse sequences: sagittal T2-weighted (T2W) fast spin-echo (FSE) fat-saturated (FS), axial T2W FSE FS, coronal short tau inversion recovery (STIR), and postcontrast axial T1-weighted (T1W) FSE FS.

### Imaging review

The four MRI sequences mentioned above were retrieved for each patient, de-identified, and then uploaded to a HIPAA-compliant cloud-based platform for image review (Ambra Health; Intelerad). Examinations were then independently reviewed by 13 fellowship-trained pediatric radiologists (M.R.A., C.G.A., J.C., A.M.E., M.M.F., M.S.G., M.L.G., R.P.G., M.K., S.V.L., M.A.R., G.R.S., A.J.T.) from eight different institutions. Their experience ranged from four to twenty-six years post-fellowship training. Imaging findings were documented by radiologists using an electronic case report (REDCap; Research Electronic Data Capture) which is presented in Supplementary Table 1. For each imaging study, reviewers also were asked to indicate whether postcontrast T1W images improved their ability to detect and characterize perianal CD compared to noncontrast T2W images alone. MRI scanner field strength and vendor information were also documented. If an abscess/collection was identified, its length in three dimensions were recorded.

Imaging feature definitions (including features taken from the modified van Assche Index [11, 12] and magnetic resonance novel index for fistula imaging in CD [MAGNIFI-CD] [13]) and classification system (Parks classification of fistulas [14] and St James’ University Hospital MRI classification of perianal fistulas [15]) descriptions were provided to all readers prior to the start of our study, although there was no formal training session, and no example cases were provided (Supplementary Table 2). All reviewers were blinded to one another as well as to clinical imaging interpretations, clinical data, laboratory data, and clinical outcomes.

### Data analysis

Continuous variables were summarized as medians and interquartile ranges (IQR), while categorical variables were summarized as counts and percentages. Assessments of inter-reader agreement were performed using Fleiss’ kappa (k) statistics as well as intraclass correlation coefficients (ICC) for categorical (e.g., present/absent) and continuous (e.g., fistula length measurements) variables, respectively. 95% confidence intervals (CI) were calculated. Inter-radiologist agreement using kappa statistics was interpreted as follows: < 0 = poor, 0–0.20 = slight, 0.21–0.40 = fair, 0.41–0.60 = moderate, 0.61–0.80 = substantial, and 0.81–1.0 = almost-perfect agreement [16]. Inter-radiologist agreement using ICC was interpreted as follows: 0–0.39 = poor, 0.40–0.59 = fair, 0.60–0.74 = good, and 0.75–1.0 = excellent [17].

All statistical analyses were performed using MedCalc Statistical Software version 22.009 (MedCalc Software Ltd.) or R (R version 4.3.1, http://www.r-project.org). A *p*-value of less than 0.05 was considered statistically significant for inference testing.

## Results

### Study sample

**Fig. 1:**
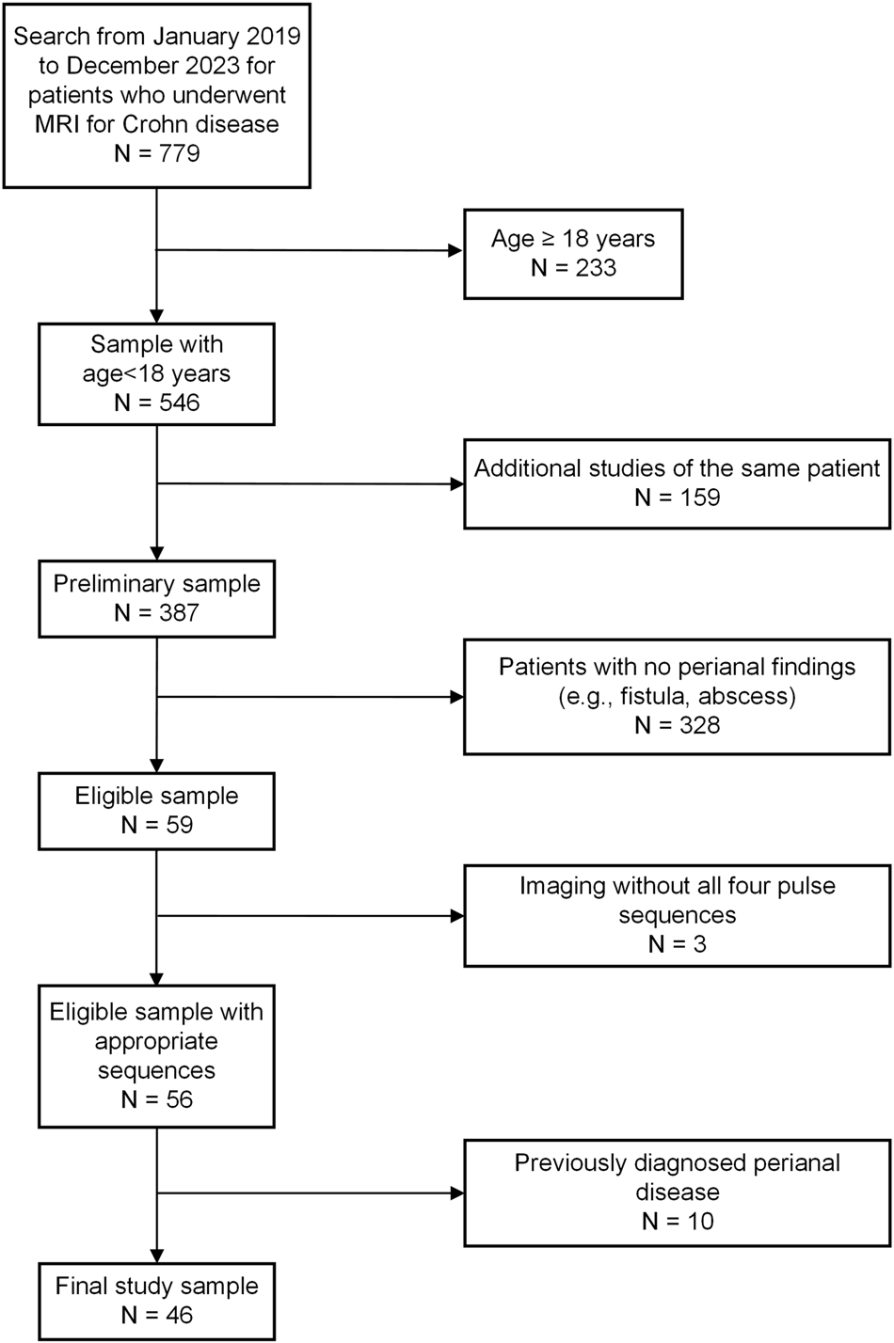
Study participant flow diagram. Boxes on the right denote excluded participants based on established inclusion and exclusion criteria

Initially, 779 MRI examinations were identified during the study period. After exclusion criteria were applied (Fig. 1), 46 subjects were included in our final study sample. Participant median age was 13.0 (IQR: 10.5 to 16.0) years; there were 35 (76%) male and 11 (24%) female patients. A participant flow diagram is shown in Fig. 1.

The majority of MRI examinations were acquired on 1.5-T scanners (*n* = 39, 85%), with the remainder performed on 3-T scanners (*n* = 7, 15%). Thirty (65%) examinations were performed on Philips Healthcare scanners, while 16 (35%) examinations were performed on GE HealthCare scanners.

### Inter-radiologist agreement

Kappa statistics and ICCs for all imaging features and classification systems evaluated are presented in Table 1.

**Table 1:** Inter-radiologist agreement for 13 pediatric radiologists in children with newly diagnosed perianal Crohn’s disease undergoing pelvic magnetic resonance imaging

Imaging features	κ / ICC (95% CI)	*p*-value
Largest dimension of abscess/collection	0.81* (0.41 to 1.00)	< 0.001
Volume of abscess/collection	0.77* (0.34 to 1.00)	< 0.001
Abscess/collection	0.52 (0.31 to 0.73)	< 0.001
Involvement of the scrotum, vagina and/or labia	0.45 (0.26 to 0.63)	< 0.001
Fistula length (< 2.5 cm, 2.5–5 cm or > 5 cm)	0.42 (0.32 to 0.53)	< 0.001
Number of fistula tracts (none, single-unbranched or complex)	0.35 (0.24 to 0.46)	< 0.001
Proctitis	0.34 (0.21 to 0.46)	< 0.001
Extension of perianal disease ﻿(absent, infralevator, horseshoe configuration, supralevator)	0.29 (0.20 to 0.37)	< 0.001
Inflammatory mass	0.29 (0.20 to 0.37)	< 0.001
Hyperintensity on T2-weighted images (primary tract and/or extensions)	0.26 (0.16 to 0.35)	< 0.001
St James’ University Hospital MRI classification of perianal fistulas	0.24 (0.17 to 0.31)	< 0.001
Hyperintensity of primary tract on postcontrast T1-weighted images	0.23 (0.13 to 0.33)	< 0.001
Parks classification of perianal fistulas	0.22 (0.13 to 0.32)	< 0.001
Dominant feature (fibrous, granulation tissue, pus)	0.21 (0.01 to 0.40)	0.039

The agreement was evaluated using Fleiss’ kappa statistics unless otherwise denoted

*CI* confidence interval, *ICC* intraclass correlation coefficient, *MRI* magnetic resonance imaging

* Agreement evaluated using ICC

Most MRI features of perianal CD had fair agreement between study radiologists. This included hyperintensity of primary tract on T2W and postcontrast T1W images, dominant feature (fibrous vs. granulation tissue vs. pus), extension of perianal disease, presence of an inflammatory mass, number of fistula tracts, presence of proctitis, Parks Classification, and St James’ University Hospital MRI Classification (κ = 0.21 to 0.35).

**Fig. 2:**
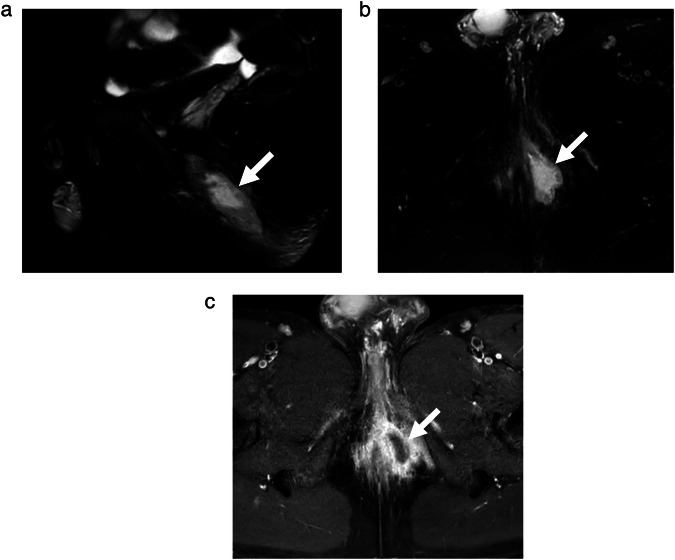
15-year-old boy with perianal Crohn’s disease. MRI pulse sequences include (**a**) sagittal T2-weighted (T2W) fast spin-echo (FSE) fat-saturated (FS), (**b**) axial T2W FSE FS, and (**c**) axial postcontrast T1-weighted (T1W) FSE FS. White arrows show a perianal peripherally enhancing fluid collection, consistent with an abscess. All 13 radiologists agreed on the presence of abscess/collection in this patient

**Fig. 3:**
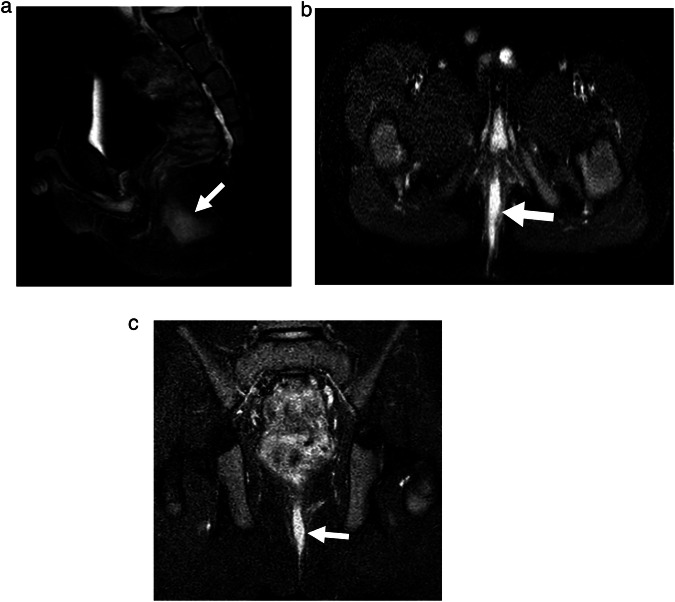
6-year-old boy with perianal Crohn’s disease. MRI pulse sequences include (**a**) sagittal T2-weighted (T2W) fast spin-echo (FSE) fat-saturated (FS), (**b**) axial T2W FSE FS, and (**c**) coronal short tau inversion recovery (STIR). White arrows demonstrate posterior midline linear hyperintense signal abnormality, consistent with a perianal fistula. When using the Parks classification, system, five radiologists described this fistula tract as trans-sphincteric, five described it as intersphincteric, and three described it as suprasphincteric

There was moderate agreement among readers for categorical fistula length (ICC = 0.42 [95% CI: 0.32 to 0.53]), involvement of scrotum, vagina and/or labia (κ = 0.45 [95% CI: 0.26 to 0.63]), and presence of an abscess/collection (κ = 0.52 [95% CI: 0.31 to 0.73]). Maximum length of abscess/collection had excellent agreement (ICC = 0.81 [95% CI: 0.41, 1.00]), while abscess/collection volume also had excellent agreement (ICC = 0.77 [95% CI: 0.34, 1.00]). Figures 2 and 3 illustrate features with high and low agreement.

There was an almost equal split (yes vs. no: 50.7% vs. 49.3%) regarding if postcontrast T1W images added value compared to noncontrast T2W images alone across all radiologists and examinations.

## Discussion

MRI is generally considered a highly accurate tool for the evaluation of perianal fistulas and abscesses in both children and adults [18, 19]. This imaging method requires high image quality with a clear depiction of anatomy as well as sufficient interpreting radiologist experience in order to achieve ideal results. Accurate MRI assessment of perianal disease is particularly important in pediatric patients with CD, as this complication may be highly morbid if not adequately treated or treated inappropriately [20, 21]. Our study has shown mostly fair agreement among pediatric radiologists when evaluating pelvic MRI features of perianal CD in children, with some imaging features demonstrating slightly better agreement. The levels of inter-radiologist agreement observed in our study help pediatric gastroenterologists and radiologists better understand the reliability of this test and areas of needed improvement.

In an adult study using four radiologists, Dane et al reported almost-perfect agreement between radiologists for the presence of a perianal fistula (κ = 0.79) or perianal abscess (κ = 0.93) [10]. Unlike our study, where all patients were known to have some form of clinical and radiologic perianal CD, in their study a smaller proportion of patients had perianal disease [10]. Furthermore, their study did not evaluate the various individual MRI features of perianal CD or specific classification systems assessed in our study [10]. Rees et al reported agreement between five pediatric radiologists for the presence of perianal complications in children and young adults with small bowel CD undergoing computed tomography enterography (CTE) and MRE and showed moderate agreement between readers (κ = 0.42) [22]. That study also did not assess the various individual MRI features of perianal CD or specific classification systems used in our study.

Rijn and colleagues evaluated inter-reader agreement for different features of the modified Van Assche index in adults and showed higher agreement than that observed in our study for number of fistula tracts (κ = 0.81), extension of perianal disease (κ = 0.65), T2W signal hyperintensity (κ = 0.65), dominant feature (κ = 0.40), and inflammatory mass (κ = 0.61) [23]. Abscess/collections (κ = 0.53) had a similar level of agreement to our study (κ = 0.52) [23]. Samaan et al also studied the modified van Assche index in adults, and their reported agreement was similar or higher than our study. These authors showed the following levels of inter-reader agreement: T2W signal hyperintensity (ICC = 0.54), inflammatory mass (ICC = 0.59), hyperintensity on postcontrast T1W images (ICC = 0.40), dominant feature of primary tract (ICC = 0.37) and extensions (ICC = 0.48) [24]. Agreement regarding the number of fistula tracts (ICC = 0.35) and presence of abscess/collection (ICC = 0.61) were similar to our study (κ = 0.35 and 0.52, respectively) [24].

Another group evaluated the MAGNIFI-CD activity index in adults with perianal CD and also reported higher agreement than that observed in our study, including number of tracts (κ = 0.76), postcontrast T1W signal hyperintensity (κ = 0.71), dominant feature (κ = 0.56), fistula length (κ = 0.85), extension (κ = 0.62), and inflammatory mass (κ = 0.64) [25]. Lower overall agreement in children compared to adults could be due to multiple reasons, including patient or technique-related factors such as age-related differences in anatomy (e.g., shorter length and smaller diameter of anal canal [26]), greater motion artifacts and lower image quality (e.g., need for a relatively larger field of view to achieve adequate signal-to-noise ratio), or radiologist-related factors such as inconsistent training in MRI feature definitions and classification systems. Imaging techniques that allow for smaller field of views with preserved signal-to noise ratios (e.g., through the use of deep learning image reconstruction algorithms or surface coils that are located closer to the anatomy of interest), and decreased motion artifacts are ideal for improving image quality and likely improving inter-reader agreement.

The Parks and St James University Hospital MRI classification systems are used by radiologists and surgeons to describe and report CD patients with perianal involvement [14, 15]. Such systems also have been used in patients to monitor response to treatment [27]. Expert panels from the United Kingdom and Europe have recommended that imaging features such as number of tracts and Parks classification system should be included in all MRI reports of patients with perianal CD [28, 29]. Based on our study, both number of tracts (κ = 0.35) and Parks classification (κ = 0.22) have only fair agreement, which may limit their clinical utility unless better inter-reader agreement can be obtained. The levels of agreement observed regarding perianal CD classification systems may, in part, reflect the fact that pediatric radiologists do not routinely clinically use or receive standardized training on such schema.

Lung et al previously reported excellent inter-reader agreement between radiologists (ICC: 0.95) when measuring fistula volume using open-source segmentation software in adults with perianal CD [30]. Subjective fistula response to biologic treatment was good (κ = 0.69) in their study. In our study, both abscess/collection volume (ICC = 0.77) and largest dimension (ICC = 0.81) had similar levels of excellent agreement, suggesting that such measurements can likely be reliably used to guide therapy. This is likely due to pediatric radiologists being more familiar with such objective assessments from their routine clinical practice.

Interestingly, on average, our readers were nearly evenly divided regarding the value of postcontrast T1W imaging compared to noncontrast T2W fat-saturated imaging alone across all examinations. Thus, our study brings into question if and when intravenous contrast material should be used. The use of intravenous contrast material adds to examination length and cost and is associated with occasional morbidity (e.g., physiologic and allergic-like contrast reactions). While further studies are needed to determine the exact value of contrast-enhanced images in children with perianal CD, the potential removal of contrast material exposure from standard protocols could have numerous benefits and deserves further investigation.

Our study has limitations. First, all MRI examinations were performed at a single, large children’s hospital using a single standardized protocol. While this limitation could potentially impact the generalizability of our results, it is notable that imaging studies were performed on MRI scanners from two different manufacturers and at two different field strengths (1.5 and 3 T), thus mirroring many clinical practices. Second, as our study was designed to evaluate inter-radiologist agreement, the ground truth for the various MRI features and classification systems assessed remains unknown. Third, we did not specifically assess radiologists’ previous familiarity with imaging feature definitions/classification schema or provide specific case-based training, although written definitions/descriptions were provided prior to imaging review. Fourth, most images were obtained in true planes (e.g., axial, coronal, and sagittal) rather than angled in-plane/orthogonal to the anal canal. Fifth, the four MRI pulse sequences used for this study employed some form of fat saturation that could potentially limit the precise evaluation of anal sphincter anatomy and that could potentially impact perianal CD classification. Sixth, while all of the readers in our study are practicing, trained pediatric radiologists and interpret these examinations in their routine clinical practices, they did not receive specialized gastrointestinal radiology training outside of their pediatric imaging fellowship, which may be another reason for the limited agreement. Finally, our study is unable to determine the impact of radiologist agreement on patient outcomes.

In conclusion, agreement between pediatric radiologists when interpreting pelvic MRI examinations for perianal CD in children is fair for most imaging features, with some imaging findings demonstrating better agreement. We believe that understanding the degree of MRI inter-reader agreement is crucial to understanding the reliability of these examinations, since interpretations may directly affect patient management (e.g., need for surgery, assessment of treatment response). MRI remains the most effective and least invasive diagnostic tool for assessing this condition in children, but additional radiologist education, more objective definitions of imaging features, and/or more objective diagnostic tools could help improve inter-radiologist agreement, although further studies are needed.

## Supplementary information

ELECTRONIC SUPPLEMENTARY MATERIAL

## Abbreviations

CD: Crohn’s disease
CI: Confidence interval
ICC: Intra-class correlation coefficient
MRI: Magnetic resonance imaging
T1W: T1-weighted
T2W: T2-weighted
