# Supplementary material for: Magnetic resonance imaging for suspected perianal Crohn's disease in children: a multi-reader agreement study
Source: Eur Radiol. 2025 Mar 23;35(9):5856–63. doi: 10.1007/s00330-025-11469-5 (PMC12350431; doi:10.1007/s00330-025-11469-5)
Supplement: Supplementary file 1 — ELECTRONIC SUPPLEMENTARY MATERIAL [file 330_2025_11469_MOESM1_ESM.pdf]

Magnetic Resonance Imaging for Suspected Perianal Crohn Disease in Children: a Multi-Reader Agreement Study

ELECTRONIC SUPPLEMENTARY MATERIAL

Supplemental Table 1: Pelvic MRI features used to assess inter-radiologist agreement in children with perianal Crohn's Disease.

FINDING	OPTION 1	OPTION 2	OPTION 3	OPTION 4	OPTION 5	OPTION 6
Number of fistula tracts	None	Single, unbranched	Complex			
Fistula length	< 2.5 cm	2.5 – 5 cm	> 5 cm			
Extension of perianal disease	Absent/mild	Pronounced				
Hyperintensity of primary tract on postcontrast T1-weighted images	Absent	Mild	Pronounced			
Hyperintensity on T2-weighted images (Primary tract and/or extensions)	Predominantly fibrous	Predominantly granulation tissue	Predominantly fluid/pus			
Dominant feature	Absent	Infralevator	Horseshoe configuration	Supralevator		
Proctitis	Absent	Present				
Involvement of the scrotum, vagina and/or labia	Absent	Present				
Inflammatory mass	Absent	Focal	Diffuse	Collection-small	Collection-medium	Collection-large
Collection	Absent	Present				
If collection present, collection length (measured in three dimensions)	_cm x _cm x _cm					
Parks Classification of Perianal Fistulas	Intersphincteric (simple)	Transsphincteric (simple if low, complex if high)	Suprasphincteric (complex)	Extrasphincteric (complex)		
St James' University Hospital MRI Classification of Perianal Fistulas	Normal appearance	Simple linear intersphincteric fistula	Intersphincteric fistula with intersphincteric abscess or secondary fistulous track	Transsphincteric fistula	Transsphincteric fistula with abscess or secondary track within the ischioanal or ischiorectal fossa	Supralevator and translevator disease
Did contrast enhanced T1-weighted MR images add value to this study (i.e. aid in detection and/or characterization of perianal disease)?	Yes	No				

Supplemental Table 2: Definitions of MRI features and classification systems provided to study radiologists prior to imaging review.

MRI Item	Score	Definition
Hyperintensity on T2-weighted images(11, 12)	Absent	No hyperintensity visible, only scar tissue
	Mild	Slight increase in signal intensity but less than nearby in-plane vessels
	Pronounced	Tract showing equal or greater signal hyperintensity than nearby in-plane vessels
Hyperintensity of primary tract on postcontrast T1-weighted images(11-13)	Absent/mild	No hyperintensity visible or slight increase in signal intensity but less than nearby in-plane vessels
	Pronounced	Tract showing equal or greater signal hyperintensity than nearby in-plane vessels
Dominant feature of primary tract and extensions(11, 12)	Predominantly fibrous	>50% of tract has a fibrotic appearance (i.e., hypointense on fat-saturated T2-weighed images)
	Predominantly filled with granulation tissue	>50% of tract is filled with granulation tissue (i.e., hyperintense on fat-saturated T2-weighted images with enhancement of contents and wall on T1-weighted postcontrast images)
	Predominantly filled with fluid or pus	>50% of tract is filled with fluid or pus (i.e., hyperintense on fat-saturated T2-weighted images with no enhancement of contents on fat-saturated T1-weighted postcontrast images [though lining of tract may enhance])
Extension(11-13)	Absent	No extension
	Infralevator	Extends upward in the ischioanal fossa but remains below the levator ani muscle
	Horseshoe configuration	Extends into the intersphincteric space on both sides of the midline
	Supralevator	Any extension in the supralevatoric space (i.e., above where the levator plate is connected to the anorectum)
Proctitis(11, 12)	Absent	Normal appearance of rectal wall
	Present	Increased wall thickness and size of mesorectal lymph nodes (>5 mm), creeping fat, increased perimural T2 signal and enhancement
Inflammatory mass(11-13)	Absent	No inflammatory mass
	Diffuse	Diffuse inflammation of surrounding tissues
	Focal	Lesion > 3 mm in diameter on T2-weighted images (but does not include linear tracts with diameter > 3 mm) with diffuse enhancement on T1-weighted post-contrast images (i.e., granulation tissue)

	Collection-small	Circumscribed cavity 3-10 mm in diameter (but does not include linear tracts with diameter > 3 mm and if present they should be excluded from the measurement of the infiltrate[d]). Hyperintense appearance on fat-saturated T2-weighted images with rim enhancement on T1-weighted postcontrast images
	Collection-medium	As defined above except diameter measures 11-20 mm
	Collection-large	As defined above except diameter measures >20 mm
Parks Classification of Perianal Fistulas(14)	Type 1: Intersphincteric (simple)	Fistula tract between the internal anal sphincter and the external anal sphincter – in the intersphincteric space
	Type 2: Transsphincteric (simple if low, complex if high)	Fistula tract crosses the external anal sphincter
	Type 3: Suprasphincteric (complex)	Fistula tract penetrates the intersphincteric space and continues over the top of the puborectalis – penetrates the levator muscle before reaching the skin
	Type 4: Extrasphincteric (complex)	Fistula tract outside the external anal sphincter – penetrating the levator muscle
St James' University Hospital MRI Classification of Perianal Fistulas(15)	Grade 0	Normal appearance
	Grade 1	Simple linear intersphincteric fistula
	Grade 2	Intersphincteric fistula with intersphincteric abscess or secondary fistulous track
	Grade 3	Transsphincteric fistula
	Grade 4	Transsphincteric fistula with abscess or secondary track within the ischioanal or ischiorectal fossa
	Grade 5	Supralevator and translevator disease
